# Transforming Growth Factor-Beta1 and Human Gingival Fibroblast-to-Myofibroblast Differentiation: Molecular and Morphological Modifications

**DOI:** 10.3389/fphys.2021.676512

**Published:** 2021-05-21

**Authors:** Guya D. Marconi, Luigia Fonticoli, Thangavelu Soundara Rajan, Paola Lanuti, Ylenia Della Rocca, Sante D. Pierdomenico, Oriana Trubiani, Jacopo Pizzicannella, Francesca Diomede

**Affiliations:** ^1^Department of Medical, Oral and Biotechnological Sciences, “G. d’Annunzio” University of Chieti-Pescara, Chieti, Italy; ^2^Department of Innovative Technologies in Medicine and Dentistry, “G. d’Annunzio” University of Chieti-Pescara, Chieti, Italy; ^3^Department of Biotechnology, Karpagam Academy of Higher Education, Coimbatore, India; ^4^Department of Medicine and Aging Sciences, “G. d’Annunzio” University of Chieti-Pescara, Chieti, Italy; ^5^Center for Advanced Studies and Technology (CAST), “G. d’Annunzio” University of Chieti-Pescara, Chieti, Italy; ^6^“Ss. Annunziata” Hospital, ASL 02 Lanciano-Vasto-Chieti, Chieti, Italy

**Keywords:** fibroblast, myofibroblast, gingival, transforming growth factor-beta1, differentiation

## Abstract

After oral mucosal injury, the healing response following specific steps that lead to wound closure and to tissue repair. Multiple cell populations are involved in this process; in particular, fibroblasts play a key role in the production of extracellular matrix (ECM). During wound healing the remodeling of ECM is a key stage to restore the tissue functionality through multifunctional fibroblast populations that are placed in the connective tissues of gingiva and periodontal ligament. Notably, a fibroblast sub-type (myofibroblast) is centrally involved in collagen synthesis and fibrillar remodeling. The present work evidenced the role of Transforming Growth Factor-beta1 (TGF-β1) to mediate human gingival fibroblasts (hGFs) differentiation into myofibroblasts derived from gingival fibroblasts (myo-hGFs). The morphological and functional features were analyzed through Confocal Laser Scanning Microscopy (CLSM), flow cytometry, and western blotting analyses. The specific markers, such as alpha-Smooth Muscle Actin (α-SMA), Vimentin, E-cadherin, β-catenin, and Smad 2/3, were modulated in myo-hGFs after the induction with TGF-β1, at different time points (24, 48, and 72 h). After 72 h of treatment TGF-β1 operates as an inducer of hGFs into myo-hGFs differentiation. We propose that TGF-β1 may promote *in vitro* the fibroblasts-to-myofibroblasts transition *via* the morphological and molecular modifications, as the induction of α-SMA, Vimentin, E-cadherin, β-catenin, and Smad 2/3.

## Introduction

Fibroblasts are implicated in several cellular events in connective tissue and are derived from embryonic mesoderm. Accordingly, they can be identified in the major part of the tissues and organs of the body linked with extracellular matrix (ECM) molecules (McAnulty, 2007).

Fibroblasts are embryologically derived from mesenchymal tissue with a variety of phenotypic entities ranging from the non-contractile fibroblast to the contractile myofibroblast. During tissue repair, fibroblasts change their normal phenotype in a contractile form called myofibroblasts, and they lose their physiological quiescent state with a slow proliferative ability (Darby and Hewitson, 2007). Granulation tissue development and contraction are important phases for wound healing. These events are described by the contribution of a specialized mesenchymal cell with morphologic and biochemical features of the fibroblast and the smooth muscle cells, defined myofibroblasts (Ko et al., 2019). Myofibroblasts can originate from connective tissue fibroblasts nearby the wound, mesenchymal stem cells, pericytes, bone marrow-derived circulating fibrocytes, and epithelial cells.

Alpha-Smooth Muscle Actin (α-SMA) is a key marker to identify the myofibroblasts; they are also able to remodel the ECM, showing the contractile ability and the capacity to strong adhere to the ECM molecules (Gabbiani, 2003). Soluble factors that stimulate myofibroblast differentiation include heparin and Transforming Growth Factor-beta1 (TGF-β1; Desmouliere et al., 1993). The differentiation stimulated by TGF-β1 of epithelial cells to α-SMA-expressing myofibroblasts is the main characteristic of kidney fibrosis (Masszi et al., 2004). The main characteristics of the epithelial-mesenchymal transition contain the prompt loss of cell-cell interactions due to the down-regulation of Zonula Occludens-1 (ZO-1) and E-cadherin, reorganization of the cytoskeleton, acquirement of spindle-like morphology, and lastly the expression of α-SMA, the distinctive feature of the myofibroblast phenotype. This event includes the interplay of a variety of TGF-β1-induced pathways, including Smad proteins (Biernacka et al., 2011), and WNT/β-catenin signaling pathway, which is correlated with fibroblast activation, fibrosis, and tissue repair (Lam and Gottardi, 2011; Lee et al., 2017).

*In vitro* and *in vivo* experiments showed the myofibroblasts capacity to produce a strong adhesive structures called fibronexus and/or focal adhesions, in particular this structure expressed the Extra Domain (ED)-A isoform of fibronectin, an abundant protein placed in the ECM that favor the collagen attachment (Dugina et al., 2001). These cells are responsible for wound closure and for the development of the collagen-rich scar. Furthermore, their existence in tissues has been recognized as a marker of progressive fibrosis (Turner and Porter, 2013). Thus, healthy tissues do not present the myofibroblasts, but myofibroblast are abundant in the wound healing proliferative phase, then at the end of wound healing process their number notably decreases. However, alterations that occur during their commitment process can be linked with fibrosis or impairment wound healing (Smith, 2018).

Using TGF-β1 stimulus, we aimed to study the role of proteins in regulating human gingival fibroblast (hGFs)-to-myofibroblast (myo-hGFs) transition in an *in vitro* model, in order to provide a starting point to study the fundamental process that regulates the physiological wound healing process. We demonstrated that TGF-β1 may induce cellular morphological modifications associated with the myofibroblastic phenotype transition, as the formation of actin stress fibers and focal adhesions. We also showed the activation of this signaling pathway that represents a key point to significant molecular events that occur during wound healing in gingival tissue.

## Materials and Methods

### Human GFs Isolation and Culture

Primary cultures of human gingival fibroblast (hGFs) were established by the explant method previously described (Mammana et al., 2019). Fragments of healthy gingival tissue were rinsed three times in Phosphate Buffered Saline (PBS, Lonza, Basel, Switzerland) solution, cut into small tissue pieces and cultured in Dulbecco’s modified Eagle’s medium (DMEM, Lonza) supplemented with 10% Fetal Bovine Serum (FBS, Lonza) and 0.1% gentamicin [10 mg/ml; Euroclone, Pero (MI), Italy] at 37°C in 5% CO_2_ atmosphere. The gingival tissue biopsies were cultured until hGFs spontaneously migrated (about 4 weeks; Cavalcanti et al., 2015). All cells were incubated in standard conditions [37°C in a humidified atmosphere of 5% (v/v) CO_2_]. Cells were observed under inverted light microscopy (Leica Microsystem, Milan, Italy), as previously described (Libro et al., 2016). All the experiments were performed with cells processed between 4 and 8 passages and each assay were performed in triplicate.

### TGF-β1 Treatment

The hGFs cells obtained by explant method were maintained in 60 mm Petri dishes in DMEM with 10% FBS and antibiotics for later collection. To induce the gingival myofibroblasts differentiation, the hGFs were incubated in DMEM with 1% FBS (Lonza), 0.1% gentamicin (10 mg/ml, Euroclone), and treated with TGF-β1 at 10 ng/ml (S.I.A.L. srl, Rome, Italy). The TGF-β1 (10 ng/ml) supplemented media was tested at 24, 48, and 72 h. Myofibroblasts phenotype was confirmed by Flow cytometry, confocal microscopy, and Western blot. The hGFs treated with TGF-β1 (10 ng/ml) were collected after 24, 48, and 72 h in order to evaluate the grade of differentiation induced by TGF-β1 with respective untreated controls. Samples were labeled as follows: CTRL (hGFs without TGF-β1) or TGF-β1 treated (hGFs with TGF-β1) at 24, 48, and 72 h.

### Myofibroblasts Determination

As previously reported, α-SMA is a specific marker associated to the fibroblast-to-myofibroblast differentiation, while vimentin is a non-specific marker (Walker et al., 2019). For this reason, we utilized α-SMA and Vimentin antibodies to determine fibroblast to myofibroblasts differentiation by flow cytometry, confocal microscopy, and Western blot analyses. In the current work, we defined fibroblast to myofibroblasts differentiation using the following criteria: the existence of the co-expression of α-SMA and Vimentin after induction with 10 ng/ml of TGF-β1 at 24, 48, and 72 h assessed by flow cytometry, confocal microscopy, and Western blot.

### Myofibroblasts Differentiation From hGFs

Human GFs were cultured in DMEM (Lonza) supplemented with 10% FBS (Lonza) and 0.1% gentamicin sulfate (10 mg/ml; Euroclone) at 37°C in 5% CO_2_ atmosphere. Cells between the fourth and tenth passages were used for the following experiments. At Passage 6 cells were seeded overnight with medium containing 1% of FBS (Lonza) and then were treated with or without TGF-β1 for 24, 48, and 72 h. Medium was removed 24 h after seeding and fibroblasts were incubated for 24, 48, and 72 h with DMEM plus with 1% FBS (Lonza) serum alone or supplemented with 10 ng/ml TGF-β1 (S.I.A.L.). Myofibroblasts phenotype was confirmed by flow cytometry, confocal microscopy, and Western blot for α-SMA, Vimentin, E-cadherin, β-catenin, and Smad 2/3. Once the obtained cell differentiation cells were collected to perform all experiments, the untreated cells were used as negative controls (CTRL).

### Flow Cytometry Phenotype Analysis of hGFs

Freshly dissociated cells were profiled using a FACSCantoII flow cytometer running with FACSDiVa software (BD Biosciences, Milan, Italy) and settings as already described (Lachmann et al., 2012). Briefly, pellets were suspended with PBS and stained with the antibodies listed in Supplementary Table 1, incubated (30 min at 4°C, in the dark). After removing the excess of each antibody, cells were suspended in PBS and acquired by flow cytometry. For Vimentin and α-SMA analyses, an indirect staining was carried out and, in these cases, after the incubation with the primary antibodies, cells were washed and pellets were incubated with 1 μl of FITC-conjugated anti-goat or anti-mouse (BioLegend, San Diego, CA, United States), respectively for 30 min at 4°C in the dark before the acquisition by flow cytometry. Each reagent listed in Supplementary Table 1 was titrated under assay conditions. Instrument performance and data reproducibility were checked using the Cytometer Setup & Tracking Module (BD Biosciences). Prior to each run the flow cytometer was cleaned according to manufacturer’s instructions and the FACSCanto SIT Flush device was routinely used to prevent carryover. Data were analyzed using FACSDiva v 6.1.3 (BD), FACSuite v1.05 (BD), and FlowJo v8.8.6 (TreeStar, Ashland, OR, United States) software. Antibody specificities were assessed using isotype matched controls and, for surface reagents, fluorescence minus one (FMO) controls (Cossarizza et al., 2019). Controls also included unstained cells and cells stained with secondary antibody only (when applicable). The Mean Fluorescence Intensity (MFI) values (i.e., α-SMA and Vimentin) were standardized by calculating the MFI ratios, obtained by dividing each MFI by the MFI of the related control (Ciccocioppo et al., 2008; Morabito et al., 2016).

### Confocal Laser Scanning Microscope Analysis

The hGFs were processed to immunofluorescence analysis as previously reported by Pizzicannella et al. (2018). Briefly the steps are as follows: fixation in paraformaldehyde solution (4%), permeabilization (10 min) in Triton X-100 (0.5%), blocking (30 min) in skimmed milk (5%). The following primary antibodies were used to incubate all samples for 2 h: anti-α-SMA (1:200; Abcam, S.I.A.L.), anti-Vimentin (1:200; Santa Cruz Biotechnology, SantaCruz, CA, United States), anti-E-cadherin (1:200; Santa Cruz Biotechnology), anti-β-catenin (1:200; Santa Cruz Biotechnology), and anti-Smad 2/3 (1:200; Santa Cruz Biotechnology). Then the following steps are necessary to conclude the staining procedure: incubation (1 h, 37°C) with secondary antibody Alexa Fluor 568 red fluorescence conjugated goat anti-mouse antibody (1:200; Molecular Probes, Invitrogen, Eugene, OR, United States), incubation (1 h, 37°C) with Alexa Fluor 488 phalloidin green fluorescent conjugate (1:200; Molecular Probes), and as last step incubation (1 h, 37°C) with TO-PRO (1:200; Molecular Probes). Samples were detected using a Zeiss LSM800 confocal system (Zeiss, Jena, Germany; Diomede et al., 2016). NIS-Elements AR imaging software (Nikon) was used to quantify the relative fluorescence intensities of α-SMA, Vimentin, E-cadherin, β-catenin, and Smad 2/3. All the experiments were repeated at least three times. Data are presented as the mean and standard error of the mean (mean ± SEM). GraphPad Prism5 statistical software was used to evaluate the differences between the considered groups; the one-way analysis of variance and then the *post-hoc* Bonferroni test were used. The value of *p* < 0.05 was set to indicate the statistical differences. Experiments were performed in triplicate.

### Western Blot Analysis

Proteins (50 μg) collected from treated and untreated hGFs were used for the subsequent electrophoreses as previously described (Marconi et al., 2019). Then nitrocellulose membranes were incubated with the following mouse monoclonal primary antibodies: β-actin (1:5000; Santa Cruz Biotechnology), anti-α-SMA (6,82 μg/ml; Abcam, S.I.A.L.), anti-Vimentin (1:500; Santa Cruz Biotechnology), anti-E-cadherin (1:500; Santa Cruz Biotechnology), anti-β-catenin (1:500; Santa Cruz Biotechnology), and anti-Smad 2/3 (1:500; Santa Cruz Biotechnology). Then the membranes were washed with TBS (supplemented with 0.1% Tween-20) and were maintained with peroxidase-conjugated secondary antibody diluted 1:1000 in 1x TBS, 5% milk, 0.05% Tween-20 for 30 min at room temperature (Manescu et al., 2016). The ECL method was used to visualize the bands. To quantify the proteins the Bio-Rad Protein Assay (Bio-Rad Laboratories, Hercules, CA, United States) was used. β-actin was used as a housekeeping protein and to normalize the obtained densitometric values.

### Statistical Analysis

Statistical analysis was performed by means GraphPad Prism 5 software. One-Way ANOVA test was used to evaluate the statistical differences followed by *post-hoc* Tukey’s multiple comparisons tests or Bonferroni test. The value of *p* < 0.05 was set as a statistically significant value.

## Results

### Morphological Analysis Through Inverted Optical Microscopy and CLSM on Primary hGFs Induced by TGF-β1

**Figure 1:**
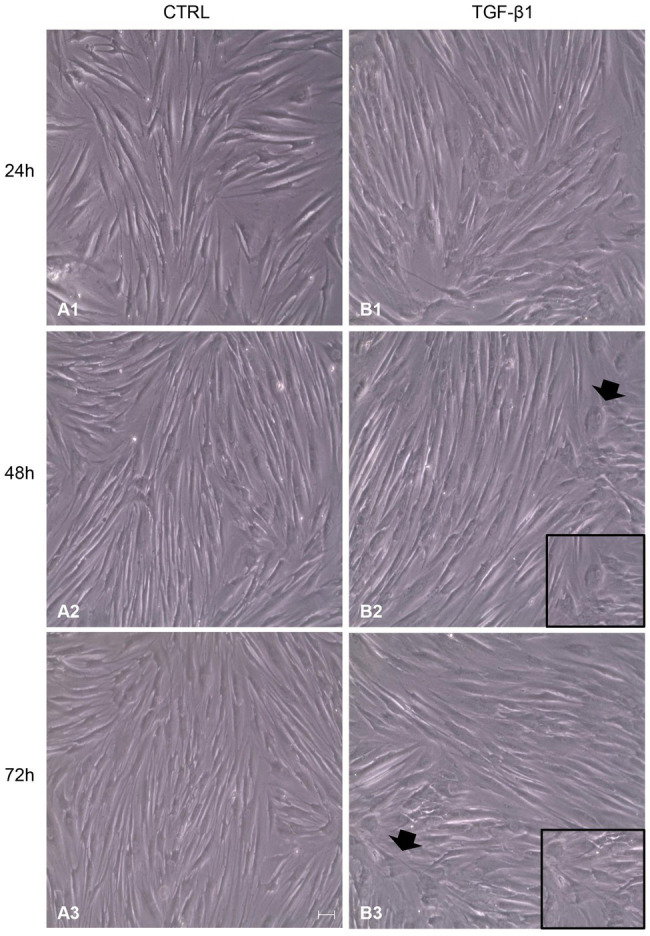
Inverted optical microscopy images of untreated human gingival fibroblasts (hGFs; CTRL; **A1**-**A3**) compared to hGFs treated with Transforming Growth Factor-beta1 (TGF-β1) (10 ng/ml) at 24, 48, and 72 h (**B1**-**B3**). Scale bar: 50 μm; magnification 10x.

**Figure 2:**
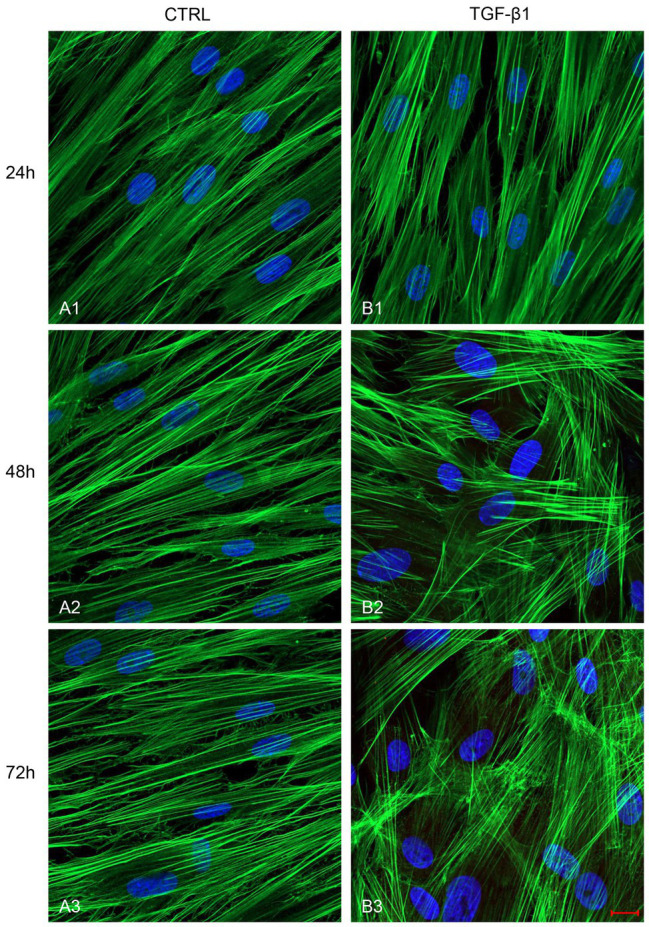
Images observed at Confocal Laser Scanning Microscopy (CLSM) of untreated hGFs (**A1**-**A3**) compared to hGFs treated with TGF-β1 (10 ng/ml) at 24, 48, and 72 h (**B1**-**B3**). Cytoskeleton actin was stained with green fluorescence; nuclei were stained with blue fluorescence. Scale bar: 20 μm; magnification 40x.

After treatment with TGF-β1 (10 ng/ml) at 24, 48, and 72 h, hGFs morphology was observed using an inverted optical microscope. Typical thin and elongated adherent fibroblasts were evidenced in the control sample while in hGFs stimulated with TGF-β1 (10 ng/ml) the cells displayed flattened shape, a typical myofibroblast phenotype, presented in the inset, (Figures 1B2,B3). Moreover, as evidenced in Figure 2, the morphology of primary hGFs has been assessed through confocal microscopy and that similar morphology changes were observed in TGF-β1-treated hGFs. The morphological modifications are more evidenced after 48 and 72 h of TGF-β1 induction than after 24 h (Figures 1, 2). To better evaluate the morphological modifications in cytoskeleton arrangement of TGF-β1-induced hGFs, immunofluorescence staining has been performed. TGF-β1 stimulated cells showed a marked expression of actin stress fibers and lost their elongated morphological aspect, as revealed by cytoskeleton actin staining by Alexa Fluour 488, at 48 and 72 h of culture when compared after 24 h (Figure 2).

### Characterization of hGFs Phenotype by Flow Cytometry

**Figure 3:**
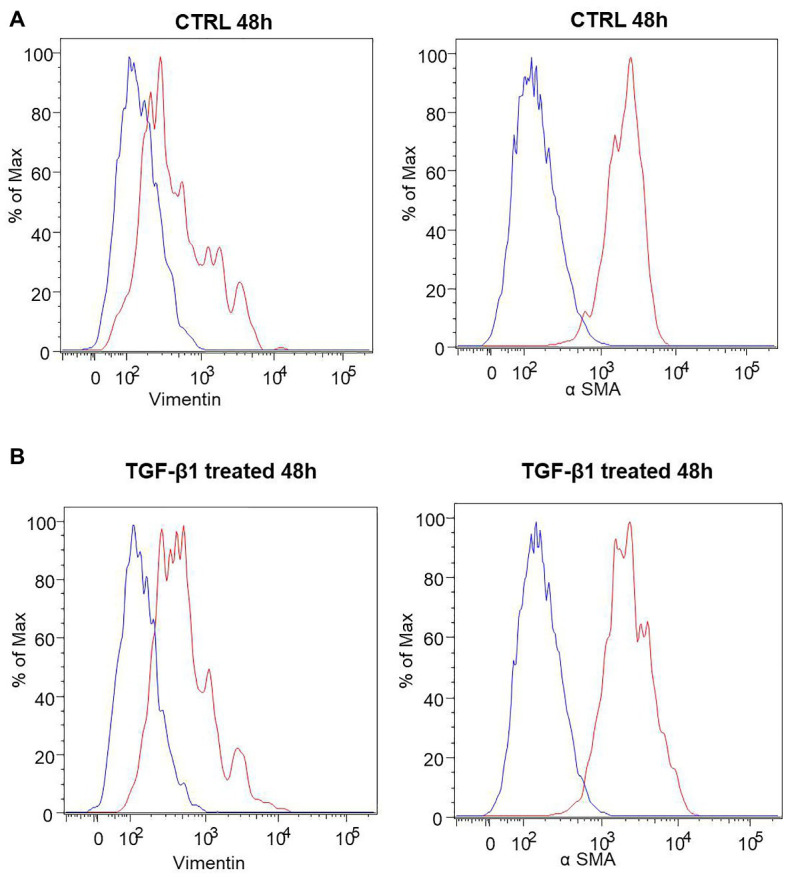
Human GFs characterization. **(A)** Flow cytometry immunophenotype analysis of untreated hGFs (CTRL) and hGFs after 48 h of TGF-β1 treatement (TGF-β1 treated). Anti-Vimentin or anti-α SMA stained cells are represented as red histograms. The related controls (cells stained with the secondary antibody alone) are represented as blue histograms. **(B)** Flow cytometry immunophenotype analysis of hGFs (CTRL) and hGFs after 72 h of TGF-β1 treatement (TGF-β1 treated). Anti-Vimentin or anti-α SMA stained cells are represented as red histograms. The related controls (cells stained with the secondary antibody alone) are represented as blue histograms.

A list of markers (Supplementary Table 1) was analyzed in hGF cells after treatment with TGF-β1 (10 ng/ml) at 24, 48, and 72 h. Among them, an increase of the expression levels of α-SMA and Vimentin, a couple of antigens associated with myofibroblast differentiation, was observed. More in detail, the treatment with TGF-β1 (10 ng/ml) at 48 and 72 h produced an increase of α-SMA levels (fold change with respect to its control sample = 16% both at 48 and 72 h) and Vimentin (fold change with respect to its control sample = 17 and 100% at 48 and 72 h, respectively) expression levels. The fold change was calculated on the basis of the MFI ratios, as described in the method section. In Figure 3 the related flow cytometry representative histograms are shown.

### Effects of TGF-β1 Treatment on the Expression of Proteins Involved in Human Gingival Fibroblasts Into Myofibroblast Differentiation Observed by Confocal Microscopy

**Figure 4:**
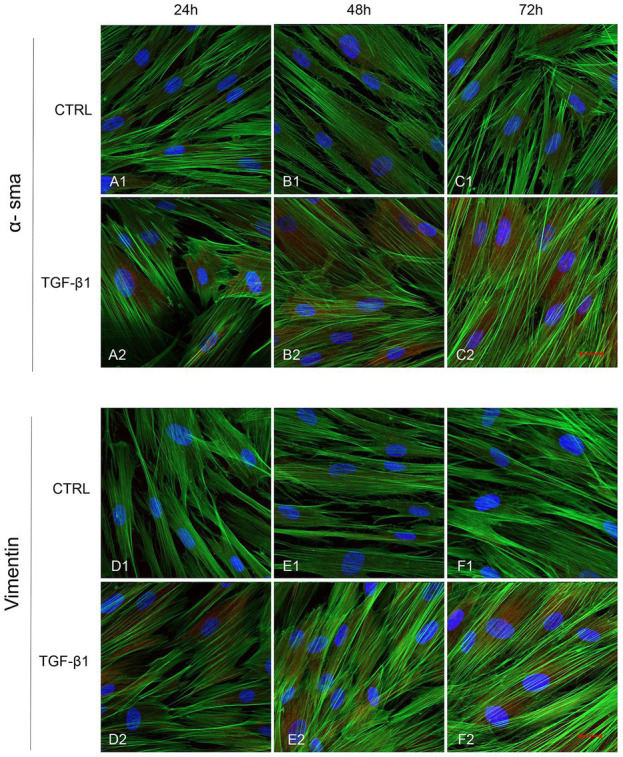
Human GFs uninduced and induced with TGF-β1 (10 ng/ml) were observed after 24, 48, and 72 h incubation. α-SMA specific marker evaluation in untreated (**A1**-**C1**) and TGF-β1 treated cells (**A2**-**C2**). Vimentin specific marker evaluation in untreated (**D1**-**F1**) and TGF-β1 treated cells (**D2**-**F2**). Red fluorescence: specific marker; green fluorescence: cytoskeleton actin; blue fluorescence: nuclei. Scale bar: 20 μm; magnification 40x.

**Figure 5:**
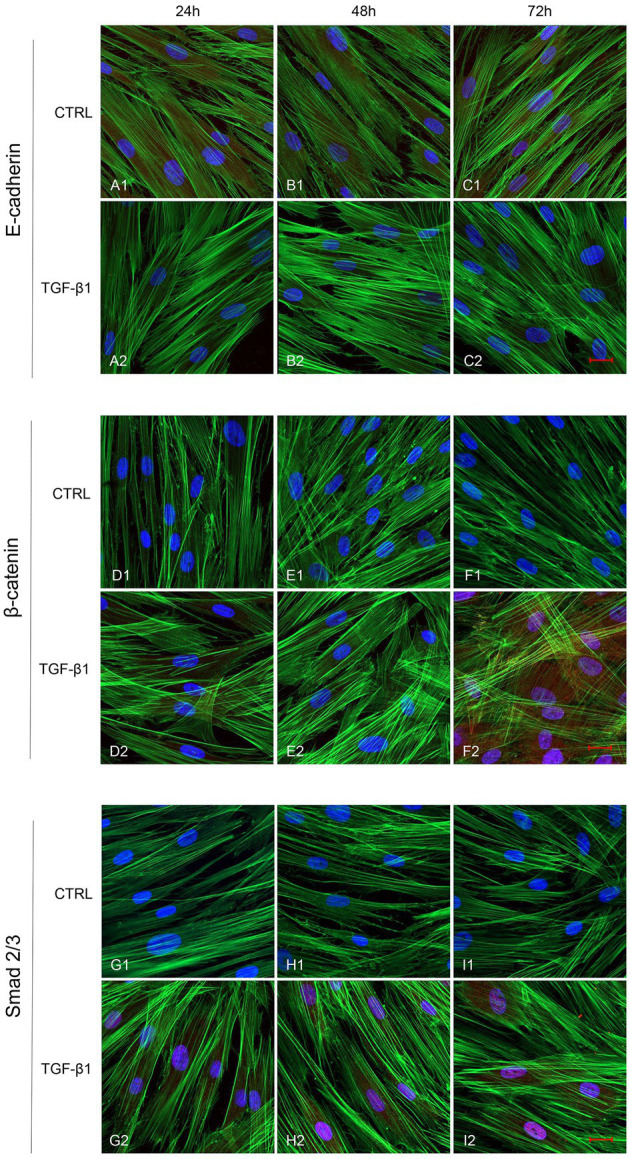
Human GFs uninduced and induced with TGF-β1 (10 ng/ml) were observed after 24, 48, and 72 h incubation. E-cadherin specific marker evaluation in untreated (**A1**-**C1**) and TGF-β1 treated cells (**A2**-**C2**). β-catenin specific marker evaluation in untreated (**D1**-**F1**) and TGF-β1 treated cells (**D2**-**F2**). Smad 2/3 specific marker evaluation in untreated (**G1**-**I1**) and TGF-β1 treated cells (**G2**-**I2**). Red fluorescence: specific marker; green fluorescence: cytoskeleton actin; blue fluorescence: nuclei. Scale bar: 20 μm; magnification 40x.

**Figure 6:**
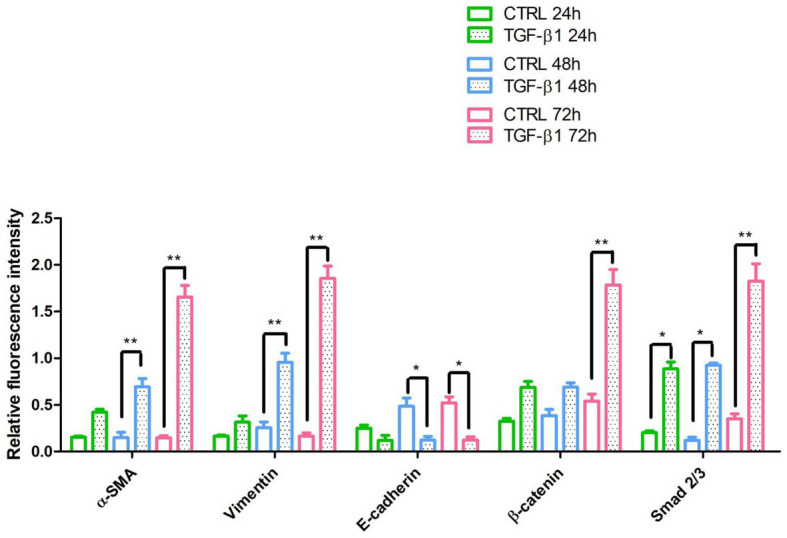
The relative fluorescent intensity of α-SMA, Vimentin, E-caderin, β-catenin, and Smad 2/3 was analyzed using NIS-Elements AR imaging software. Data are presented as the mean of 10 measurements ± SD. ^*^p < 0.05; ^**^p < 0.01.

Initial phase of myofibroblast differentiation is controlled mostly by TGF-β1, which represents the typical inducer of myofibroblast differentiation. Immunofluorescence staining was executed to establish the expression of markers involved during myofibroblasts differentiation. α-SMA, a key marker of differentiated myofibroblast, showed an increased expression in time-dependent manner in hGFs induced with TGF-β1 (10 ng/ml) at 24, 48, and 72 h (Figures 4A2–C2) compared to the untreated samples (Figures 4A1–C1), observed by confocal microscopy. Vimentin, a marker of naive fibroblasts, evidenced a positive expression in time-dependent manner in hGFs stimulated with TGF-β1 (10 ng/ml) at 24, 48, and 72 h (Figures 4D2–F2) compared to the untreated cells (Figures 4D1–F1). The same trend is displayed for β-catenin, the intracellular binding partner of E-cadherin; the cells showed an increased expression of β-catenin in time-dependent manner in hGFs treated with TGF-β1 (10 ng/ml) at 24, 48, and 72 h (Figures 5D2–F2) compared to the untreated cells (Figures 5D1–F1). The same pattern is also shown for Smad 2/3, the major transcriptional effector of the TGF-β1 signaling cascade, which is involved in control of α-SMA expression; the cells exhibited an augmentation in expression in time-dependent manner in TGF-β1-induced cells (10 ng/ml) at 24, 48, and 72 h (Figures 5G2–I2) compared to the untreated cells (Figures 5G1–I1). On the contrary, E-cadherin evidenced a decreased expression in time-dependent manner in hGFs treated with TGF-β1 (10 ng/ml) at 24, 48, and 72 h (Figures 5A2–C2) in comparison with the cells without the TGF-β1 induction (Figures 5A1–C1), observed by confocal microscopy. The relative fluorescence intensites have been reported in Figure 6 to quantify the different expression of α-SMA, Vimentin, E-caderin, β-catenin, and Smad 2/3 in all considered conditions.

### Western Blot Analysis

**Figure 7:**
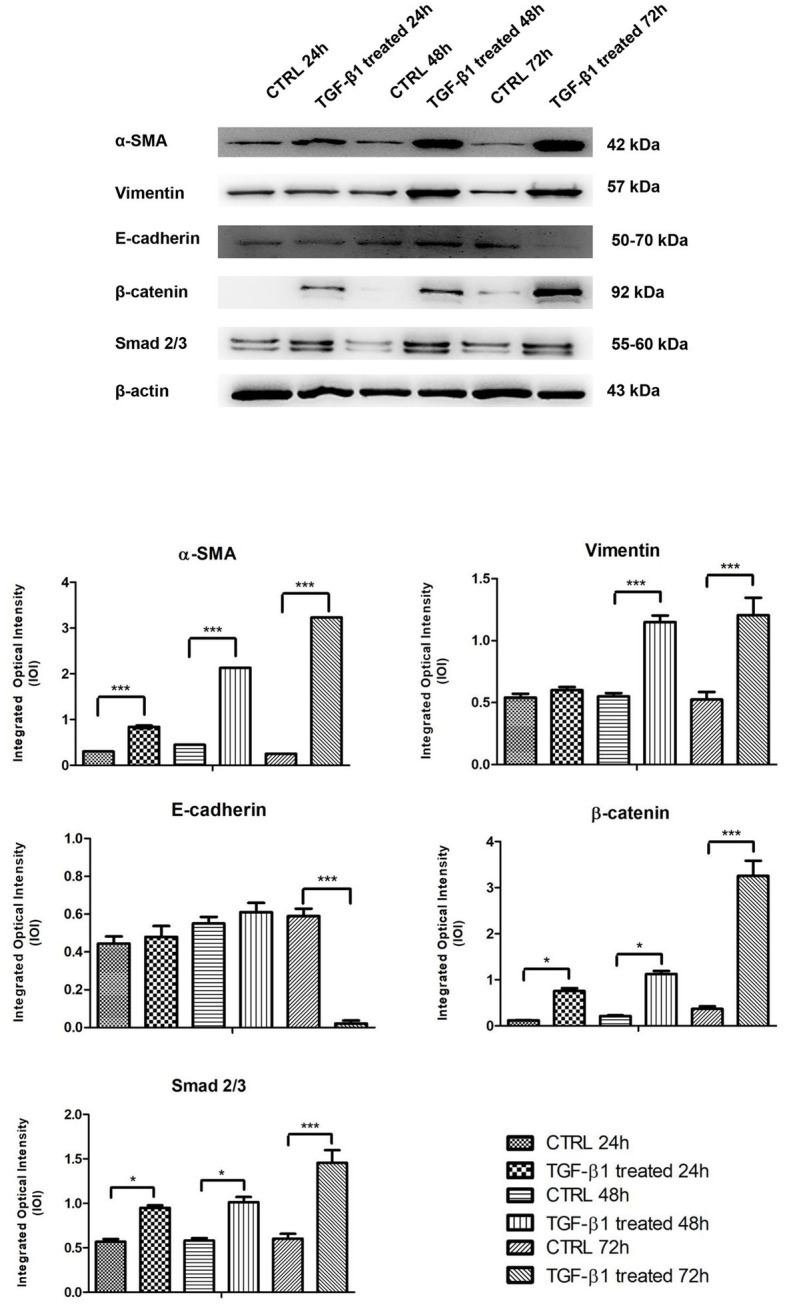
Western blotting analysis of α-SMA, Vimentin, E-cadherin, β-catenin, and Smad 2/3 expression in primary hGFs treated with TGF-β1 (10 ng/ml) after 24, 48, and 72 h. Each membrane was probed with β-actin antibody to verify protein loading consistency. The given Western blot is the representative image of three different experiments. Histograms represent densitometric measurements of proteins bands expressed as Integrated Optical Intensity (IOI) mean of three separate experiments. The error bars on the graphs show standard error of the mean (±SEM). ^*^p < 0.01; ^***^p < 0.001.

The proteins involved in myofibroblast transdifferentiation were evaluated in hGFs with or without the induction with TGF-β1 (10 ng/ml) at different time points: 24, 48, and 72 h. The image analysis of the western blot specific bands showed an overexpression of α-SMA protein after TGF-β1 (10 ng/ml) treatment in time-dependent manner in comparison to the untreated samples (Figure 7). The higher expression of α-SMA protein is reported at 72 h compared to the other samples at 24 and 48 h treated and untreated with TGF-β1 (10 ng/ml). A similar trend is exhibited for β-catenin and Smad 2/3; the proteins evidenced an increase of the expression in time-dependent manner after the induction of TGF-β1 (10 ng/ml) when compared to the untreated samples. β-catenin and Smad 2/3 displayed a significant augmentation after 72 h of induction compared to the other samples at 24 and 48 h treated and untreated with TGF-β1 (10 ng/ml). Furthermore, Vimentin reported no significant change in its expression with or without the stimulation with TGF-β1 (10 ng/ml) after 24 h, while a higher expression of the protein is evidenced on hGFs treated with TGF-β1 (10 ng/ml) compared to the untreated sample after 48 and 72 h. In parallel, E-cadherin was also investigated, and its expression evidenced a higher level of protein expression on hGFs induced with TGF-β1 (10 ng/ml) compared to the untreated sample after 24 and 48 h; instead, after 72 h of induction its expression decreased respected to the untreated sample. Taken together, these results established that the treatment with TGF-β1 (10 ng/ml) induced the fibroblasts to myofibroblasts differentiation at 24, 48, and 72 h.

## Discussion

In the wound healing remodeling phase, myofibroblasts, a “special” fibroblast sub-type, are involved in the production and rearrangement of proteins contained in the wound extracellular matrix (Gabbiani et al., 1978). In particular the role of myofibroblasts is evidenced during physiologic and pathologic process; in particular, they are fundamental to matrix reorganization and tissue contraction (Desmouliere et al., 1993; Papaccio et al., 2017, 2018). During the physiological process that occurs during tissue restoration, they are present for a short time and then disappear by means of the apoptosis mechanism. Despite this, the chronic presence and persistent activity of myofibroblasts is attributed to several fibrotic pathologies in tissues of different organ and apparatus as liver, kidney, and lung (Darby et al., 2016). Myofibroblasts were detected in the granulation tissue, and their crucial role in wound contraction was reported (Chitturi et al., 2015). Recent works have provided a more intricate definition of these cells: myofibroblasts are a cell population with specific phenotype features showing the presence of parallel bundles of fibers at the cytoplasmic level, a jagged nucleus with evident nucleoli, other than an high represented Golgi apparatus (Gabbiani et al., 1978; Eyden, 2005). Myofibroblasts can originate from several cell types, including resident fibroblasts, fibrocytes, adipocytes, monocytes, mesenchymal cells, and epithelial/endothelial cells (van Caam et al., 2018). During the physiological process of tissue restoration, myofibroblasts disappear *via* apoptosis mechanism or they are able to differentiate in fibroblasts at the end of healing process, but if myofibroblasts remain in the wound tissue may result in the scar production (Ko et al., 2019). In recent years, the role of myofibroblasts has captured the attention of researchers for their possible application in regenerative medicine. Myofibroblasts are commonly involved in the production of ECM components, such as collagens, glycoproteins, and proteoglycans, and the augmentation of matrix elements such as laminin, glycosaminoglycans, and hyaluronic acid. In gingival tissue during the wound healing process, myofibroblasts originate from resident fibroblasts, which are stimulated by cytokines and growth factors (Rout et al., 2002).

Transforming growth factor-β (TGF-β) is produced in the early stages of wound healing, produced by activated macrophages and neutrophils, which act as chemotactic and mitogenic factor to gingival and periodontal ligament cells, other than to be involved in the myofibroblast differentiation (Kuru et al., 2004; Yi et al., 2016). As reported by Smith et al. (2006), TGF-β1 was able to bring some modifications in the adhesive properties of hGFs and may increase the expression levels of several integrins (Rout et al., 2002; Smith et al., 2006). TGF-β1 is also considered an important factor in fibrosis regulation and in the induction of the transition of fibroblast phenotype to myofibroblast *in vitro* by means of the Smad 3-dependent pathway (Desmouliere et al., 1993; Fang and Svoboda, 2005; Willis et al., 2006; Hinz et al., 2012).

Cho et al. (2018) reported the role of TGF-β1 in differentiation of human cardiac fibroblasts to myofibroblasts in the context of myocardial pathology (Cho et al., 2018). The acquisition of myofibroblast phenotype is associated with the expression of specific markers, as α-SMA, and in the same time the lacking of the characteristic epithelial markers, such as E-cadherin and ZO-1 (Willis et al., 2006). E-cadherin down-regulation enhances, while β-catenin knockdown inhibits, α-SMA expression. The implication of β-catenin in the process of epithelial-myofibroblast transition, fibrogenesis, and α-SMA expression was reported (Charbonney et al., 2011). Furthermore, it is largely described that α-SMA and Vimentin are markers for mesenchymal factors (Li et al., 2019).

On this basis, in the present work we have explored the molecular and morphological modifications induced by TGF-β1 during the differentiation of gingival myofibroblasts in a time-dependent manner (24, 48, and 72 h of stimulation). This process was observed by measuring the specific markers as α-SMA, Vimentin, E-cadherin, β-catenin, and Smad 2/3 that are implicated in the phenotype and functional switch. Our findings, obtained through confocal microscopy, evidenced that the expression levels of α-SMA, Vimentin, β-catenin, and Smad 2/3 were significantly increased in primary hGFs treated with TGF-β1 in a time-dependent manner. Furthermore, to confirm the phenotype switch from hGFs into myo-hGFs, E-cadherin expression was assessed. E-cadherin is a protein related to the cell-cell adhesion which participates in the development of cell junctions; its main function was recognized in the establishing and preservation of epithelial cell polarity (Zheng et al., 2016).

Furthermore, E-cadherin is related to the expression of α-SMA, binding the β-catenin at the intracytoplasmatic level. The role of β-catenin was demonstrated in the cellular adhesion and is strictly related to tissue restoration and fibrosis, playing a pivotal role in the activation of fibroblast transdifferentiation through WNT/β-catenin signaling pathway (Liu et al., 2019).

During the formation of pathological scarring, myofibroblasts are able to modulate the biochemical and biophysical microenvironment that lead to the alterations of tissue architecture; when their function appears deregulated the tissue fibrosis is associated with organ damage (Fintha et al., 2019). Our results showed that the exposure of primary hGFs to TGF-β1 was related with an increase of α-SMA and cytoskeleton. In fact, at the basis of the contractile properties of myofibroblasts there is the high expression of α-SMA and its incorporation in the cytoskeleton stress fibers (O’Connor and Gomez, 2014). Myofibroblasts under the stimulation of TGF-β1 can rearrange the protein and fibers of ECM other than stimulate the contraction collagen placed at the extracellular level (Gabbiani, 1988; Smith et al., 2019). In detail, the immunofluorescence staining showed a remarkable downregulation of E-cadherin, an epithelial marker, in hGFs treated with TGF-β1 compared to untreated samples. We assessed using confocal microscopy that the TGF-β1 in time-dependent manner affected myofibroblast differentiation. Our observation that the TGF-β1 exposure at 72 h suggested the higher myofibroblast differentiation showing a significant expression of α-SMA, Vimentin, β-catenin, and Smad 2/3 and a negative expression for E-cadherin. At the same time, our immunofluorescence data have been validated by Western blotting analysis. α-SMA, Vimentin, β-catenin, and Smad 2/3 were upregulated in the TGF-β1 treated hGFs compared to the untreated samples; E-cadherin was found to be downregulated after 48 and 72 h of TGF-β1 treatment compared to the untreated hGFs. Taken together these findings revealed that the TGF-β1 treatment led to a differentiation of primary hGFs to a myofibroblast-like phenotype. These results improved our understanding of the molecular and morphological modifications happening during the primary gingival fibroblast to myofibroblast differentiation. Moreover, the development of this *in vitro* model could be a starting point to study the pathologies related with reduced or additional deposition of ECM.

In the current study, we reported that TGF-β1 are able to promote the induction of the myofibroblastic phenotype through enhance the expression of α-SMA, Vimentin, β-catenin, and Smad 2/3 in hGFs. These modifications at phenotypical level included the presence of focal adhesion and the rearrangement of cytoskeleton actin fibers organization. Taken together, all morphological and molecular modifications in gingival fibroblasts could represent an important step in the commitment to a myofibroblastic phenotype during physiological and pathological steps that lead to a tissue restoration.

Future studies are necessary to define the cellular and molecular mechanism of these cell populations and provide more knowledge in the differentiation process, which are dramatically important for the definition of novel strategies in the regeneration of oral tisues. Moreover, to translate the *in vitro* model to *in vivo* context mechanical signaling pathways, including mechano-transduction, cell contractility, and regulation of matrix rigidity, should be taken into account. Fibroblasts and their special sub-type play a fundamental role during gingival and periodontal ligament wound healing when applied as medical devices, as in other tissues.

## Data Availability Statement

The raw data supporting the conclusions of this article will be made available by the authors, without undue reservation.

## Ethics Statement

The studies involving human participants were reviewed and approved by Ethical Committee, School of Medicine and Life Sciences, “G. d’Annunzio” University, Chieti, Italy. The patients/participants provided their written informed consent to participate in this study.

## Author Contributions

GDM and LF have contributed equally to this work and share first authorship. JP and FD have contributed equally to this work and share senior authorship. All authors contributed to the article and approved the submitted version.

### Conflict of Interest

The authors declare that the research was conducted in the absence of any commercial or financial relationships that could be construed as a potential conflict of interest.
