# Supplementary material for: Transforming Growth Factor-Beta1 and Human Gingival Fibroblast-to-Myofibroblast Differentiation: Molecular and Morphological Modifications
Source: Front Physiol. 2021 May 21;12:676512. doi: 10.3389/fphys.2021.676512 (PMC8176099; doi:10.3389/fphys.2021.676512)
Supplement: Supplementary file 1 [file Table_1.DOCX]

| **Supplementary Table 1.** Reagent list | | | | |
| --- | --- | --- | --- | --- |
| *Reagent* | *Fluorochrome/Reagent* | *Vendor* | *Catalogue Number* | *Volume per test (μl)* |
| CD13 | FITC | Ancell | ANC-162-040 | 1 µl |
| CD29 | PE | Ancell | ANC-178-050 | 1 µl |
| CD34 | PerCP | BD Biosciences | 345803 | 10 µl |
| CD117 | APC | BD Biosciences | 333233 | 5 µl |
| Sox2 | AlexaFluor 488 | BD Biosciences | 560301 | 5 µl |
| Vimentin | goat polyclonal+ anti-goat-FITC | Santa Cruz | sc-7558 | 1 µl |
| α-SMA | mouse monoclonal + anti-mouse-FITC | Santa Cruz | sc-53142 | 5 µl |
| α-SMA = Alpha-smooth muscle actin; APC = Allophycocyanin; FITC = Fluorescein isothiocyanate; PE = R-phycoerythrin; PerCP = Peridinin-Chlorophyll-Protein. | | | | |
